# Direct Biomarkers of Microbial Translocation Correlate with Immune Activation in Adult Zambians with Environmental Enteropathy and Hepatosplenic Schistosomiasis

**DOI:** 10.4269/ajtmh.17-0365

**Published:** 2017-08-28

**Authors:** Patrick Kaonga, Evans Kaimoyo, Ellen Besa, Kanekwa Zyambo, Edford Sinkala, Paul Kelly

**Affiliations:** 1 Tropical Gastroenterology and Nutrition Group, The University of Zambia School of Medicine, Lusaka, Zambia;; 2 Department of Internal Medicine, The University of Zambia School of Medicine, Lusaka, Zambia;; 3 Department of Biological Sciences, The University of Zambia, Lusaka, Zambia;; 4 Department of Internal Medicine, The University Teaching Hospital, Lusaka, Zambia;; 5 Department of Internal Medicine, The University Teaching Hospital, Lusaka, Zambia;; 6 Blizard Institute Barts and The London School of Medicine, Queen Mary University of London, London, United Kingdom

## Abstract

Microbial translocation is a poorly understood consequence of several disorders such as environmental enteropathy (EE) and hepatosplenic schistosomiasis (HSS). Herein, we compared biomarkers of microbial origin and immune activation in adults with these disorders and in healthy controls. A cross-sectional study was conducted in participants with EE recruited from Misisi compound, Lusaka, Zambia; HSS patients and healthy controls from the University Teaching Hospital, Lusaka. Plasma lipopolysaccharides (LPSs) was measured by limulus amoebocyte lysate assay, plasma 16S ribosomal RNA (16S rRNA) gene copy number was quantified by quantitative real-time polymerase chain reaction, Toll-like receptor ligand (TLRL) activity by QUANTI-Blue detection medium, and cytokines from cell culture supernatant by Cytometric Bead Array. In univariate analysis LPS, 16S rRNA gene copy number, and TLR activity were all high and correlated with each other and with cytokines tumor necrosis factor-α (TNF-α), interleukin-6 (IL-6), IL-10, and IL-4 secreted by the RAW-Blue cells. After controlling for baseline characteristic, biomarkers of microbial translocation in blood were predictors of TNF-α, IL-6, and IL-10 activation in cell culture supernatant from EE participants and HSS patients but not in healthy controls. TLR activity showed the strongest correlation with TNF-α. These data provide correlative evidence that microbial translocation contributes to systemic cytokine activation in two disorders common in the tropics, with total TLR ligand estimation showing the strongest correlation with TNF-α (*r* = 0.66, *P* < 0.001).

## INTRODUCTION

Microbial translocation (MT) is the passive movement of microbes and/or their products from the gut into mesenteric lymph nodes and other sterile sites. It is common in environmental enteropathy (EE), a subclinical condition hypothesized to be acquired through repeated exposure to fecal–oral contamination.^1^ EE is characterized by loss of barrier function, chronic intestinal inflammation, MT, and chronic immune activation.^2^ MT can be measured by detection of direct biomarkers in plasma such as lipopolysaccharide (LPS),^3^ 16S ribosomal RNA (16S rRNA) gene copy number,^4^ or other pathogen-associated molecular patterns (PAMPs). Hepatosplenic schistosomiasis (HSS) refers to the major complication of chronic infection with *Schistosoma mansoni*, *Schistosoma japonicum*, *Schistosoma intercalatum*, or *Schistosoma mekongi*. One of the major consequences of HSS disease is portal hypertension, which is associated with esophageal varices or gastric varices or both. Variceal bleeds which result from increased portal pressure may lead to MT.^5^ In certain parts of rural Zambia prevalence of *S. mansoni* has reached 77%.^6^

A recent study in Zambia reported elevated LPS-binding protein (LBP) and soluble CD14 (sCD14), surrogate biomarkers of MT as well as soluble CD163 (sCD163), a biomarker of inflammation in HSS patients compared with healthy controls.^7^ The optimal measure of MT is not yet established. Previous studies have measured direct biomarkers of MT using LPS^8,9^ and 16S rRNA gene copy number^10,11^ without detecting other microbial components which could lead to chronic immune activation and if not controlled may results into microcirculatory dysfunction, septic shock, tissue damage, and mortality.^12^ We set out to compare three different approaches to measurement of MT in blood samples, by measuring LPS, bacterial DNA, and Toll-like receptor ligand (TLRL) activity, which detects almost all possible PAMPs in plasma including lipoglycans, lipoproteins, lipoteichoic acids, and peptidoglycans.

## MATERIALS AND METHODS

### Samples and sources.

We conducted a cross-sectional study in Misisi compound, which is an unplanned settlement in the southern part of Lusaka city, Zambia, where EE is prevalent.^13,14^ Eighty-one community adult participants between the ages of 18 and 60 years were recruited after door-to-door sensitization and focus group discussions resulting in their respective consent to participate in the study. Those involved in other studies, or with helminth infection found in stool, pregnant, those on antibiotics or nonsteroidal anti-inflammatory drugs within a month were excluded. EE was confirmed by endoscopy using confocal laser endomicroscopy, followed by binocular microscopy immediately after endoscopy, then formal morphometry of duodenal biopsies after endoscopy, though the degree of EE varied significantly. One hundred and five adult patients recruited from University Teaching hospital with HSS underwent endoscopy. HSS patients were defined by positive serology for schistosomiasis which was done by microwell enzyme-linked immunosorbent assay (ELISA) (Scimedx Corporation, Denville, NJ), hematemesis and/or splenomegaly, and esophageal/gastric varices. The exclusion criteria for HSS patients were inability to give consent, alcohol dependency which was ascertained by questions only, cirrhosis by ultrasound examination, or being seropositive for hepatitis B or C viral infection. We enrolled 65 EE participants, 86 HSS patients, and 40 healthy controls that underwent endoscopic examination and were found to have a normal gut with no HSS disease. The baseline characteristics of study participants are shown (Table 1).

**Table 1:** Baseline characteristics of study participants

Variable	EE group (*N* = 65)	HSS patients (*N* = 86)	Healthy controls (*N* = 41)	*P* value
Sex (M:F)*	22:43	34:52	20:21	0.41
Age	29 (24, 43)	40 (30, 51)	32 (25, 38)	0.01
Education, secondary or more (*n*, %)*	25 (39)	33 (38)	15 (42)	0.80
BMI (kg/m^2^)	22.9 (20.5, 27.6)	22.7 (21.4, 26.9)	23.6 (21.2, 28.3)	0.20
Hb (g/dL)	13.3 (12.5, 14.9)	9.7 (6.4, 11.5)	13.6 (11.2, 18.3)	< 0.001
Platelet count (×10^9^/L)	232 (200, 280)	121 (87, 137)	189 (143, 230)	< 0.001
White cell count (×10^9^/L)	4.2 (2.8, 4.9)	2.5 (2, 4.1)	4 (4, 7)	< 0.001
HIV seropositive	14 (22)	0 (0)	0 (0)	< 0.001
CD4 count (cells/μL)	516 (350, 694)	–	–	–

BMI = body mass index; EE = environmental enteropathy; Hb = hemoglobin; HIV = human immunodeficiency virus; HSS = hepatosplenic schistosomiasis; F = female; M = male.

* χ^2^ was used; percentages are in parentheses. For continuous variables median (interquartile range) are shown.

### Blood collection.

Blood was collected from all enrolled participants and centrifuged to extract plasma within 1 hour and stored at −80°C until use.

### Measurement of LPS.

To measure LPS in plasma samples we used the Limulus amebocyte lysate (LAL)-chromo assay (Associates of Cape Cod Incorporated, Falmouth, MA), according to the manufacturer’s instructions.

### Real-time quantitative polymerase chain reaction for measurement of bacterial 16S rRNA in blood.

DNA was isolated from blood using the QIAamp DNA Mini Kit(Qiagen, Hilden, Germany) according to the manufacturer’s instructions. The primer pair used was 16S F519 5′-CAGCAGCCGCGGTAATAC-3′ and 16S R785 5′-TGGACTACCAGGGTATCTAATCC-3′, which is specific for conserved DNA sequences encoding the 16S rRNA gene.^15^ The real-time quantitative polymerase chain reaction (PCR) mix consisted of 2 × SYBR green PCR mix 0.4 μL of a mixture of both forward and reverse primers at a final concentration of 0.2 mM, 5 μL of DNA, and 4.6µL endotoxin free water to a final volume of 20μL. A plasmid DNA with known copy number of template was serially diluted by 10-fold from 1 × 10^6^ to 1 × 10^0^ copies and used to generate a standard curve which was used to calculate copies of 16S rRNA genes; the lowest detection limit was 1 × 10^2^ copies/µL. PCR was performed using a Rotor gene 6000 thermal cycler (Corbett, North West, Australia); the amplification profile consisted of initial denaturation at 95°C for 15 minutes followed by 40 cycles at 95°C for 15 seconds, 60°C for 20 seconds, and 72°C for 45 seconds. For the specific identification of 16S rRNA gene, a melt-curve analysis was conducted by heating PCR products from 65°C to 99°C with continuous acquisition and products were subjected to a ramping profile of 0.2°C/seconds. C_*t*_ values of more than 35 cycles were declared as not detected.

### TLR ligand assay using RAW-Blue™ mouse macrophage cell line.

The Raw-Blue mouse macrophage cell line (InvivoGen, San Diego, CA) carries a chromosomal integration of a secreted embryonic alkaline phosphatase (SEAP) reporter gene construct inducible by transcription factors NF-κB and AP-1. Cells were grown on Dulbecco’s Modified Eagle medium as recommended by the supplier, and cell viability was assessed using trypan blue.^16^ To detach confluent cells, they were scraped and resuspended in RAW-Blue test media plated on flat bottom 96-well plate at a density of 1 × 10^5^/mL in 180 µL of the test medium and incubated with 20 µL of plasma for 24 hours at 37°C in 5% CO_2_ humid atmosphere. After incubation, 50 µL of the cell culture supernatant was incubated with 150 µL of QUANTI-Blue™ detection reagent in a flat bottom 96-well culture plate and then incubated for 1 hour at 37°C. TLR ligand activity (SEAP activity) was determined using a spectrophotometer at 630 nm.

### Analysis of cytokines in RAW-Blue cell culture supernatants.

After 24 hours incubation, cytokines were measured in the cell culture supernatant using a Cytometric Bead Array (CBA) mouse Th1/Th2/Th17 kit (Becton Dickinson, Heidelberg, Germany). The cytokines measured were tumor necrosis factor-α (TNF-α), interleukin (IL)-6 (IL-6), IL-10, IL-4, IL-2, IL-17, and interferon–γ (IFN-γ). Immune activation was defined as cytokine secretion ratio of 2 or more compared with the control supernatant. After acquiring samples on a flow cytometer, FCAP Array™ software (San Jose, CA) was used to analyze results.

### Measurement of other plasma biomarkers of host response to MT.

We used commercially available ELISA kits to measure plasma biomarkers of host immune response to MT (sCD14; [R and D Systems, Inc., Abingdon, UK]); immune activation (C-reactive protein [CRP; R and D Systems]) and soluble (CD163 [sCD163; R and D Systems]).

### Data analysis.

To compare baseline characteristics among EE, HSS patients, and healthy controls, the χ^2^ test was used for categorical variables and the Kruskal–Wallis test for continuous variables because the D’Agostino–Pearson test showed the data was not normally distributed. Multivariate multiple regression models were constructed to determine whether direct biomarkers were predictors of cytokines while controlling for baseline characteristics with the probability of removal in the final model set at *P* < 0.20 (20%) and interpreted using regression coefficients and confidence intervals. To understand the relationships among biomarkers and between biomarkers and cytokines, Spearman’s rank correlation was used. All statistical analyses were done using GraphPad Prism, version 5.01 (GraphPad Software Inc., La Jolla, CA) and STATA, version 13 (Stata Corp, College Station, TX). For all statistical tests, a *P* value < 0.05 was considered statistically significant.

### Ethical consideration.

This study was reviewed and approved by the University of Zambia Biomedical Research and Ethics Committee. Informed written consent was obtained from the participants before participation in the study.

## RESULTS

### Baseline characteristics of study participants.

HSS patients were slightly older than participants in the other two groups, but were more likely to have lower hemoglobin, white cell count, and platelets (Table 1). Participants with EE had a substantial prevalence of human immunodeficiency virus (HIV) (22%; *P* < 0.001) compared with HSS patients and the healthy comparison group of whom none had infection. Other characteristics such as sex, body mass index, education level, and alcohol consumption were comparable (Table 1).

### Direct biomarkers of MT in EE group, HSS patients, and healthy controls.

**Figure 1.**
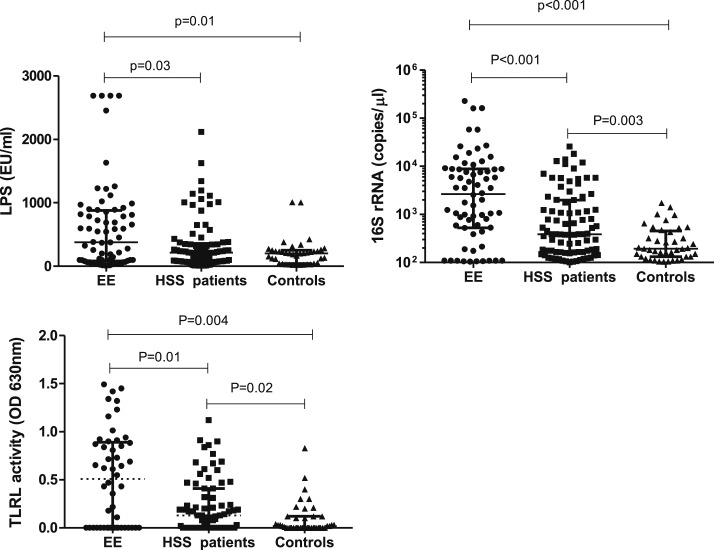
(**A**) Comparison of lipopolysaccharide, (**B**) 16S rRNA gene copy number, and (**C**) Toll-like receptor ligand activity among environmental enteropathy group, hepatosplenic schistosomiasis patients, and healthy controls. Kruskal–Wallis test was used to compare across the groups and Dunns posttest was used to compare all pairs. The results are shown with significance where applicable (*P* < 0.05).

To compare biomarkers of microbial origin among EE, HSS patients, and healthy controls, we measured LPS, 16S rRNA, and TLR ligand. In univariate analysis, the EE group had significantly higher LPS in plasma (median 378.9 [82.7–879.5]) compared with HSS patients (213.1 [77.2–358.3] EU/mL) or healthy controls (202.3 [43.2–251.1] EU/mL) but no difference between HSS patients and healthy controls (Figure 1A). As LPS is only present in gram-negative bacteria, real-time quantitative PCR was used to quantify 16S rRNA gene copy number in blood. 16S rRNA copy number was higher in the EE group (median 2,651 [529–8,779] copies/µL) than the HSS patients (387 [165–1,990] copies/µL; *P* < 0.001) or the healthy controls (193 [132–455] copies/µL; *P* < 0.001) (Figure 1B). The RAW-Blue mouse macrophage cell line was stimulated with plasma to detect total PAMPs activity in plasma. TLRL activity was significantly higher in the EE group (median 0.49 [0.0–0.8] optical density [OD]) than in HSS patients (0.13 [0.0–0.8] OD; *P* = 0.01) or the healthy controls (0.02 [0.0–0.12] OD; *P* = 0.004) (Figure 1C).

#### Cytokines in EE group, HSS patients, and healthy controls measured from cell culture supernatants.

**Figure 2.**
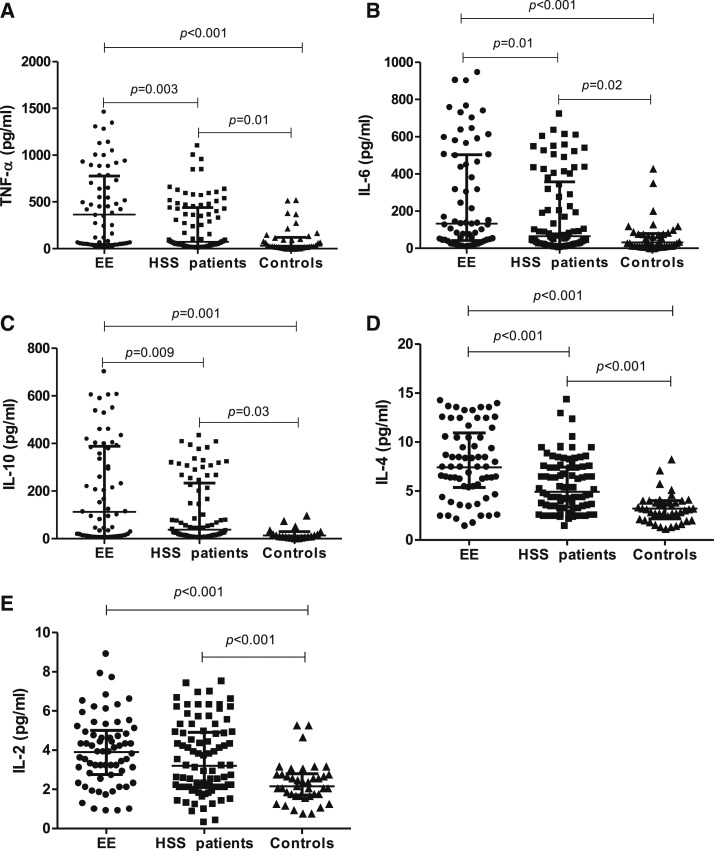
Comparison of cytokines among environmental enteropathy group (*N* = 65), hepatosplenic schistosomiasis patients (*N* = 86), and healthy controls (*N* = 40) measured in cell culture supernatant. Differences in (**A**) tumor necrosis factor-α (**B**) interleukin (IL)-6, (**C**) IL-10, (**D**) IL-4, and (**E**) IL-2. Kruskal–Wallis test was used to compare across the groups and Dunns posttest was used to compare all pairs. The results are shown with significance where applicable (*P* < 0.05).

Since MT is thought to drive immune activation,^3,17–19^ we further analyzed the profile of cytokine secretion by the macrophage cells in the EE group and HSS patients compared with healthy controls. To determine whether there were differences in immune activation among groups, we measured TNF-α, IL-6, IL-10, IL-4, IL-2, IFN-γ, and IL-17 produced by stimulated RAW-Blue mouse macrophages.^20^ Immune activation was defined as a ratio of 2 or more to the secretion under control conditions. In univariate analysis, TNF-α, IL-6, IL-10, and IL-4 were significantly higher in the EE group compared with HSS patients but there was no difference in IL-2. All cytokines were elevated in the EE and HSS groups compared with healthy controls (Figure 2A–E).

### Measurement of host response to MT.

**Figure 3.**
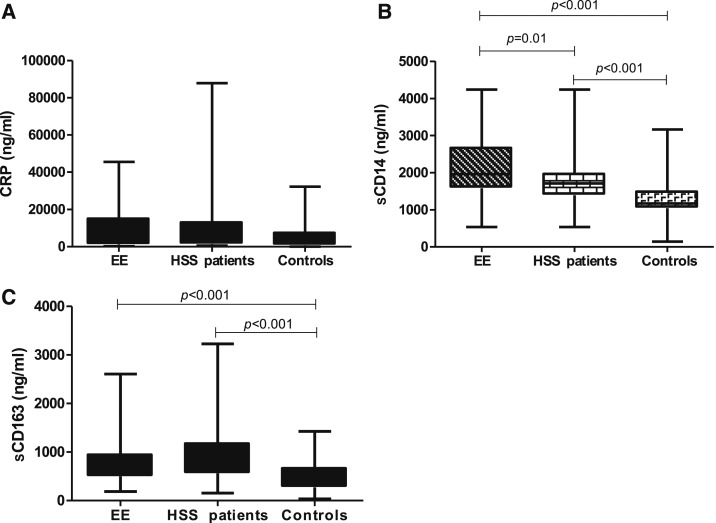
Comparison of plasma biomarkers among environmental enteropathy group (*N* = 65), hepatosplenic schistosomiasis patients (*N* = 86), and healthy controls (*N* = 40) measured in plasma. Differences in (**A**) C-reactive protein, (**B**) soluble CD14, and (**C**) soluble CD163. Kruskal–Wallis test was used to compare across the groups and Dunns posttest was used to compare all pairs. The results are shown with significance where applicable (*P* < 0.05).

To assess the host response to MT, we analyzed plasma levels of CRP, sCD14, and sCD163. CRP is an acute phase response protein, which is elevated for a short time produced mainly in the liver in response to IL-6.^21^ We found no difference among all groups in CRP levels (Figure 3A). To assess MT indirectly, we measured the surrogate markers sCD14 and sCD163 in plasma. The EE group had significantly higher sCD14 in plasma (median 1,959 [1,582–2,669] ng/mL) than HSS patients (1,712 [1,389–1,964] ng/mL) or healthy controls (median 1,170 [1,045–1,489] ng/mL; *P* < 0.001) (Figure 3B; *P* < 0.001). sCD163 did not differ between EE group and HSS patients (Figure 3C) but higher levels were found in either group than in healthy controls (*P* < 0.001; Figure 3C).

#### Relationship between biomarkers of microbial origin and cytokines from the cell culture supernatant.

We next explored the relationship between biomarkers of microbial origin in plasma and cytokines from the cell culture supernatant. With EE and HSS groups combined, TNF-α was positively and strongly correlated with LPS (*r* = 0.61; *P* < 0.001), 16S rRNA copy number (*r* = 0.49; *P* < 0.001), and TLRL activity (*r* = 0.66; *P* < 0.0001) (Supplemental Figure 1A, C, and E). In the healthy controls, TNF-α was not significantly correlated with LPS (*r* = 0.03), 16S rRNA copy number (*r* = 0.22), or TLRL activity (*r* = 0.31) (Supplemental Figure 1B, D, and F). For correlations in the individual groups, EE group had stronger correlation of TNF-α with LPS, 16S rRNA gene copy number, and TLRL activity compared with either HSS patients or healthy controls (data not shown). Generally, combining EE group and HSS participants resulted in increased and more significant correlation of all activated cytokines with direct biomarkers of MT compared with a single group or with healthy controls (Supplemental Figures 1, 2, and 3).

### Relationship between biomarkers of microbial origin and biomarkers of host response to MT.

In an attempt to explain the differences in biomarkers of microbial origin between EE group or HSS patients and healthy controls, we analyzed the correlation with biomarkers of host response to MT.^22^ When groups were analyzed separately, the EE group had generally more significant correlation than HSS patient and healthy controls (Table 2), and when both EE and HSS groups were combined the correlations were stronger. There were no significant correlations in the healthy control group (Table 2).

**Table 2:** Correlation matrices showing relationships between biomarkers of microbial origin and biomarkers of host response to microbial translocation

Variables	LPS	16S rRNA	TLRL	CRP	sCD14	sCD163
EE group (*N* = 65)						
LPS	1.0000					
16S rRNA	0.5292**	1.0000				
TLRL	0.4376**	0.6171**	1.0000			
CRP	0.0167	0.0477	0.0667	1.0000		
sCD14	0.3734*	0.0577	0.2907*	0.0708	1.0000	
sCD163	0.1154	0.1398	0.2876*	0.0722	0.2704*	1.0000
HSS patients (*N* = 86)						
LPS	1.0000					
16S rRNA	0.5163**	1.0000				
TLRL	0.3392*	0.6254**	1.0000			
CRP	0.1550	0.1391	0.1737	1.0000		
sCD14	0.1070	0.0157	0.2622*	0.0632	1.0000	
sCD163	0.1134	0.0996	0.2131	0.0076	0.1285	1.0000
Healthy controls (*N* = 40)						
LPS	1.0000					
16S rRNA	0.0219	1.0000				
TLRL	0.2093	0.0658	1.0000			
CRP	0.1104	0.2218	0.0756	1.0000		
sCD14	0.1896	0.0615	0.0440	0.0476	1.0000	
sCD163	0.0752	0.0018	0.1217	0.1261	0.1329	1.0000
EE group and HSS patients combined (*N* = 151)						
LPS	1.0000					
16S rRNA	0.5231**	1.0000				
TLRL	0.6104**	0.6404**	1.0000			
CRP	0.0407	0.2704*	0.2308*	1.0000		
sCD14	0.3922**	0.1693	0.3112*	0.10125*	1.0000	
sCD163	0.2751*	0.1077	0.3126*	0.0541	0.3582**	1.0000

CRP = C-reactive protein; EE = environmental enteropathy; LPS = lipopolysaccharide; 16S rRNA = 16S ribosomal RNA gene; TLRL = Toll-like receptor ligand; sCD14 = soluble CD14; sCD163 = soluble CD163. Two-tailed correlations: * *P* < 0.05, ** *P* < 0.01.

**Table 3:** Multivariate multiple regression analysis of relationship between cytokines and direct biomarkers of microbial translocation

	EE (*N* = 65)		HSS patients (*N* = 86)		Healthy controls (*N* = 40)	
	*B*	95% CI	*B*	95% CI	*B*	95% CI
TNF-α						
LPS	0.04	0.03 to 0.14*	0.06	0.03 to 0.17*	0.02	−0.09 to 0.13
16S rRNA	0.004	0.002 to 0.007*	0.006	0.001 to 0.01*	0.003	−0.007 to 0.01
TLRL	11.3	6.6 to 15.4**	6.1	1.76 to13.9*	3.6	−0.95 to 8.45
IL-6						
LPS	0.006	0.01 to 0.09*	0.09	−0.008 to 0.17	0.02	−0.02 to 0.15
16S rRNA	0.04	0.0009 to 0.008*	0.01	0.002 to 0.02*	0.007	−0.001 to 0.02
TLRL	7.7	2.11 to 9.98**	4.5	1.1 to 12.3*	1.4	−0.5 to 17.3
IL-10						
LPS	0.04	0.009 to 0.71*	0.03	−0.008 to 0.06	0.02	−0.011 to 0.05
16S rRNA	0.004	0.004 to 0.02*	0.002	0.0009 to 0.008*	−0.002	−0.003 to 0.004
TLRL	1.96	0.95 to 4.9*	1.4	1.1 to 3.52*	0.64	−2.2 to 3.5
IL-4						
LPS	−0.003	−0.02 to 0.01	0.001	−0.002 to 0.003	0.0002	−0.0003 to 0.0007
16S rRNA	0.002	−0.003 to 0.008	−0.001	−0.002 to 0.001	−0.0001	−0.00003 to 0.00006
TLRL	1.2	−1.6 to 4.1	−3.1	−9.5 to 3.2	−0.08	−0.22 to 0.07
IL-2						
LPS	−0.001	−0.007 to 0.005	−0.002	−0.01 to 0.007	0.002	−0.01 to 0.008
16S rRNA	−0.005	−0.01 to 0.01	−0.006	−0.007 to 0.0009	−0.0001	−0.008 to 0.001
TLRL	0.46	−0.75 to 1.67	−1.5	−4.1 to 3.7	−0.02	−0.1 to 0.08

CI = confidence interval; CRP = C-reactive protein; EE = environmental enteropathy; HSS = hepatosplenic schistosomiasis; IL = interleukin; LPS = lipopolysaccharide; TLRL = Toll-like receptor ligand; TNF-α= tumor necrosis factor-α; sCD14 = soluble CD14; sCD163 = soluble CD163; WBC = white blood cell; 16S rRNA = 16S ribosomal RNA gene. Data are given as regression coefficient (*B*) and 95% CI. *R*^2^ is 0.526 for the EE model, 0.382 for the HSS model, and 0.040 for the healthy controls model. Outcome variables were log-transformed and reported back on the original scale of measurement.

* *P* < 0.05 significant values different from reference category adjusted for all explanatory variables in the model, age, hemoglobin, platelet, WBC, and HIV.

** *P* < 0.01 significant values different from reference category adjusted for all explanatory variables in the model, age, hemoglobin, platelet, WBC, and HIV.

### Multivariable analysis.

We constructed multivariate multiple regression models taking LPS, 16S rRNA copy number, and TLRL activity as independent variables and cytokines as dependent variables while controlling for baseline characteristics. In the EE group, a good model fit was obtained (*R*^2^ = 0.526, *F* = 47.53, *P* < 0.001) which predicted TNF-α, IL-6, and IL-10. In the HSS group, a less impressive but still significant fit was obtained (*R*^2^ = 0.382, *F* = 22.43, *P* = 0.002); of note in this model LPS did not statistically predicted activation of IL-6 and IL-10. In healthy controls, no satisfactory model was obtained (*R*^2^ = 0.040, *F* =1.03, *P* = 0.38) (Table 3).

## DISCUSSION

There is mounting evidence that MT leads to immune activation in many different disorders, including EE^2^ and HSS^7^; it is also a central issue in critical care. It is also difficult to measure. In our study, we asked whether direct biomarkers of MT in plasma correlate with immune activation in EE and HSS patients. LPS, present only in gram-negative bacteria, 16S rRNA gene copy number, and TLR ligand (total PAMPs) concentrations in plasma all correlate with immune activation in EE and HSS patients. All of these direct biomarkers of MT were higher in EE individuals compared with HSS patients or healthy controls and were strongly correlated with each other. The levels of direct biomarkers in plasma correlated with biomarkers of the host response to MT such as sCD14 and CD163. In our previous data, we found elevated LPS in EE^2^ and higher sCD14 and sCD163 in HSS patients compared with healthy controls.^7^ Other studies in chronic HIV infection^23,24^ suggested that LPS and bacterial DNA are not the only TLRLs that can lead to immune activation. In our study, we used in vitro stimulation of RAW-Blue macrophage cells to detect total PAMPs (but not flagellin) to detect other possible biomarkers other than LPS and 16S rRNA that might be involved in MT.

We acknowledge that nearly one quarter of the EE group was HIV positive, but severity of enteropathy in HIV-infected adults is almost indistinguishable (with the exception of crypt depth) from that of uninfected adults with EE.^25^ Permeability (measured by lactulose:rhamnose ratio) is increased only in acquired immune deficiency syndrome. We excluded cryptosporidiosis and other related opportunistic infection by stool examination in all participants, and in any case diarrhea in the past 2 weeks was an exclusion criterion. In previous work, we observed that plasma LPS concentrations did not differ in HIV-seronegative and -seropositive adults.^2^ We consider that HIV infection is unlikely to have explained our findings. Previous studies have reported increased 16S rRNA gene copies in the plasma of HIV-infected patients due to HIV enteropathy which cause compromised intestinal barrier function,^24,26^ whereas others have found no difference between HIV-positive patients and HIV-negative people.^27,28^

MT followed by immune activation has also been reported in other disorders such as inflammatory bowel diseases (IBDs) such as Crohn’s disease^29^ and Ulcerative colitis,^22^ in animal models such as nonhuman primates with chemically induced colitis,^30^ and in a mouse model where features of EE were triggered by diet and specific microbial exposure.^31^ Circulating CD163 has been reported as a biomarker of Kupffer cell activation,^32^ whereas others have suggested it as a biomarker of tissue homeostasis and repair involved in immune modulation.^33^ Elevated CD163 has been reported in EE^2^ and HSS patients,^7^ and in this study it was higher in the EE group compared with HSS patients or controls and was correlated with direct biomarkers of MT. The innate immune system responds to LPS through TLR4 expressed by macrophages and monocytes. Macrophages sense LPS through CD14 which together with other coreceptors such as LBP and MD2 protein binds it,^12,23,34^ resulting in NF-κb induction and subsequent production of pro-inflammatory cytokines.^35^ We report elevated levels of sCD14 in both EE group and HSS patients compared with healthy controls providing further evidence of the host response to MT. CRP is an acute phase protein produced in response to pro-inflammatory cytokines such as IL-6.^36^ We found no difference in CRP among the groups suggesting that it may be a less appropriate biomarker of the activation observed in response to MT.^37,38^

RAW-Blue cells express all human TLRs except TLR5. We report here the use of TLRL activity to quantify PAMPs in plasma of individuals with EE and HSS patients. TLRL activity has been quantified in stimulated RAW-Blue mouse macrophage cells in diverse contexts together with the pro-inflammatory cytokines such as TNF-α, IL-6, and IL-2.^20,39–41^ There are reports that elevated TNF-α leads to reduction in expression of tight junction proteins, such as claudins, resulting in increased MT.^42,43^ Claudin-2 has been specifically identified as one of the claudin proteins, which is upregulated in inflammatory diseases.^42,44^ We also measured several cytokines secreted directly by the RAW-Blue cells. TNF-α is produced by a number of cells including macrophages in response to inflammatory processes.^45^ In our study, elevated TNF-α seems to correlate more closely with MT than other cytokines. TNF is known to induce an enteropathy.^46^ As TNF-α can also modulate tight junction proteins, it is possible that MT might be exacerbated by increased levels of circulating TNF-α just as it impairs intestinal barrier function in Crohn’s dieases.^47,48^ We consider it unlikely that an effect of TNF on tight junctions would alone be sufficient to permit MT as this would probably require a larger, cellular defect in the intestinal barrier.^29^

We found higher levels of IL-6 in EE than in HSS patients or healthy controls and it correlated with biomarkers of MT. In a mouse model, treatment with a monoclonal antibody to IL-6 resulted in reduced intestinal permeability and reduced the expression of claudin-2 suggesting that suppression of IL-6 promotes intestinal barrier integrity.^49^ In IBDs, it has been suggested that IL-6 increases tight junction permeability through the mitogen-activated protein kinase/extracellular signal-regulated kinase and phosphoinositide 3-kinase pathways in intestinal epithelial cells by stimulating the expression of channel-forming claudin-2.^50^ Other studies have reported that increased IL-6 correlates with diseases severity in Crohn’s disease.^51^ We found that IL-10 was higher in the EE group compared with HSS patients or healthy controls. IL-10 is an immunomodulatory cytokine with potent anti-inflammatory properties and IL-10 treatment in IBDs has shown successful results in many mouse models.^52,53^ In human, a double-blind, placebo-controlled study was conducted to investigate the efficacy and safety of IL-10 treatment in patients with Crohn’s disease. The overall findings showed only trivial and nonsignificant clinical improvement.^54^ In a macaque model, a study was set out to characterize the pathogenesis of HIV-mediated enteropathy and demonstrated increased production of IL-10 was accompanied by disruption of epithelial barrier as evidenced by loss of ZO-1 and was associated with up regulation of mucosal TNF-α and IFN-γ suggesting that IL-10 was unable to turn down inflammatory response.^55^ Perhaps surprisingly, although studies have reported IL-10 inhibits secretion of pro-inflammatory cytokines,^56^ others have reported that IL-10 has the ability to stimulate T cells, B cells, and NK cells which can result in establishment of inflammation.^57^ MT could lead to immune activation followed by generalized and persistent chronic inflammation and immune activation as reported elsewhere,^25^ but a full explanation of the role of individual cytokines is not yet available in EE and HSS patients.

In conclusion, we found higher levels of plasma LPS, 16S rRNA, and TLRL in individuals with EE compared with HSS patients or healthy control. Plasma sCD14 and sCD163 were also elevated in EE compared with HSS patients or controls. In both EE and HSS patients, the correlation of direct biomarkers with immune activation measured by TNF-α, IL-16, and IL-10 was significant. These data are in line with a model that biomarkers of microbial origin in the gastrointestinal tract move across a compromised intestinal barrier in EE and HSS patients, inducing systemic immune activation.

## Supplementary Material

Supplemental Figure.
