# Supplementary material for: Direct Biomarkers of Microbial Translocation Correlate with Immune Activation in Adult Zambians with Environmental Enteropathy and Hepatosplenic Schistosomiasis
Source: Am J Trop Med Hyg. 2017 Aug 28;97(5):1603–10. doi: 10.4269/ajtmh.17-0365 (PMC5817780; doi:10.4269/ajtmh.17-0365)
Supplement: Supplementary file 1 [file tpmd170365.SD1.pdf]

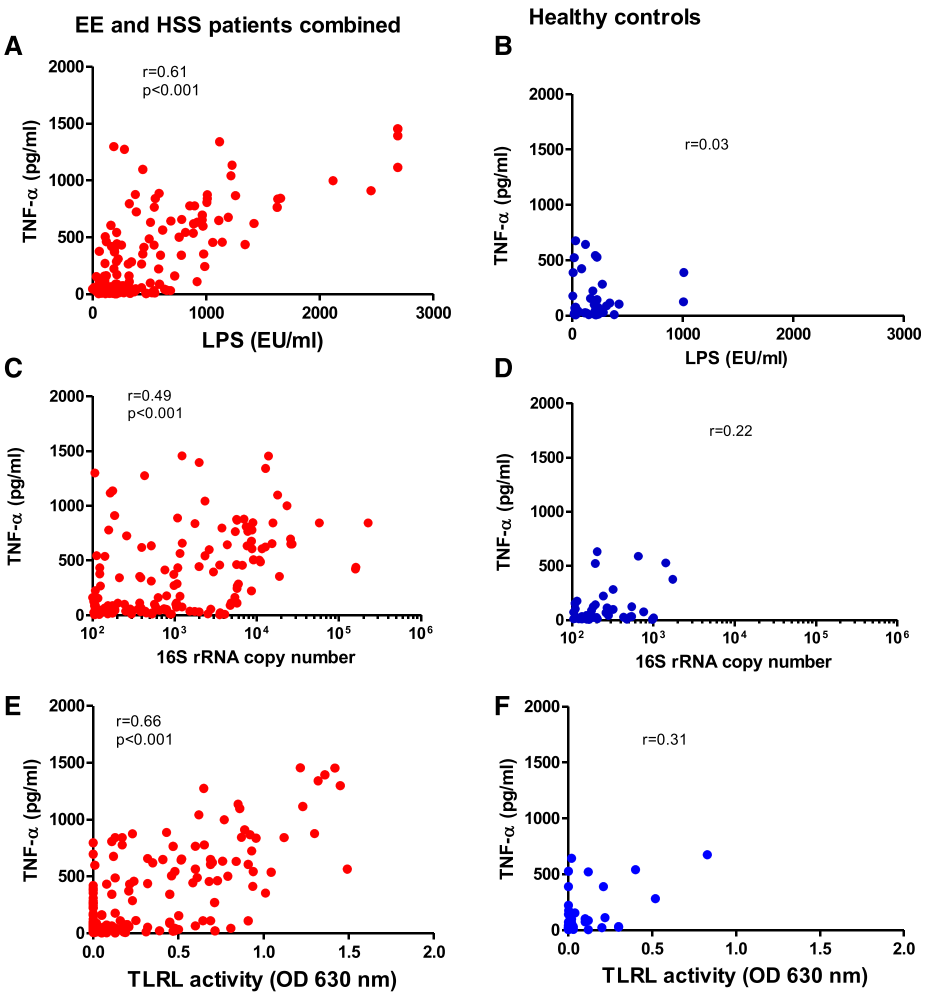

Supplemental Figure 1. Relationship between tumor necrosis factor-α (TNF-α) and direct biomarkers of microbial translocation. Environmental enteropathy and hepatosplenic schistosomiasis patients combined correlations between TNF-α and lipopolysaccharide (LPS) (Panel **A**), TNF-α and 16S rRNA copy number (Panel **C**) and TNF-α and Toll-like receptor ligand (TLRL) activity (Panel E) and in healthy controls between TNF-α and LPS (Panel **B**), TNF-α and 16S rRNA copy number (Panel **D**) and TNF-α and TLRL activity (Panel **F**). Spearman correlations are shown.

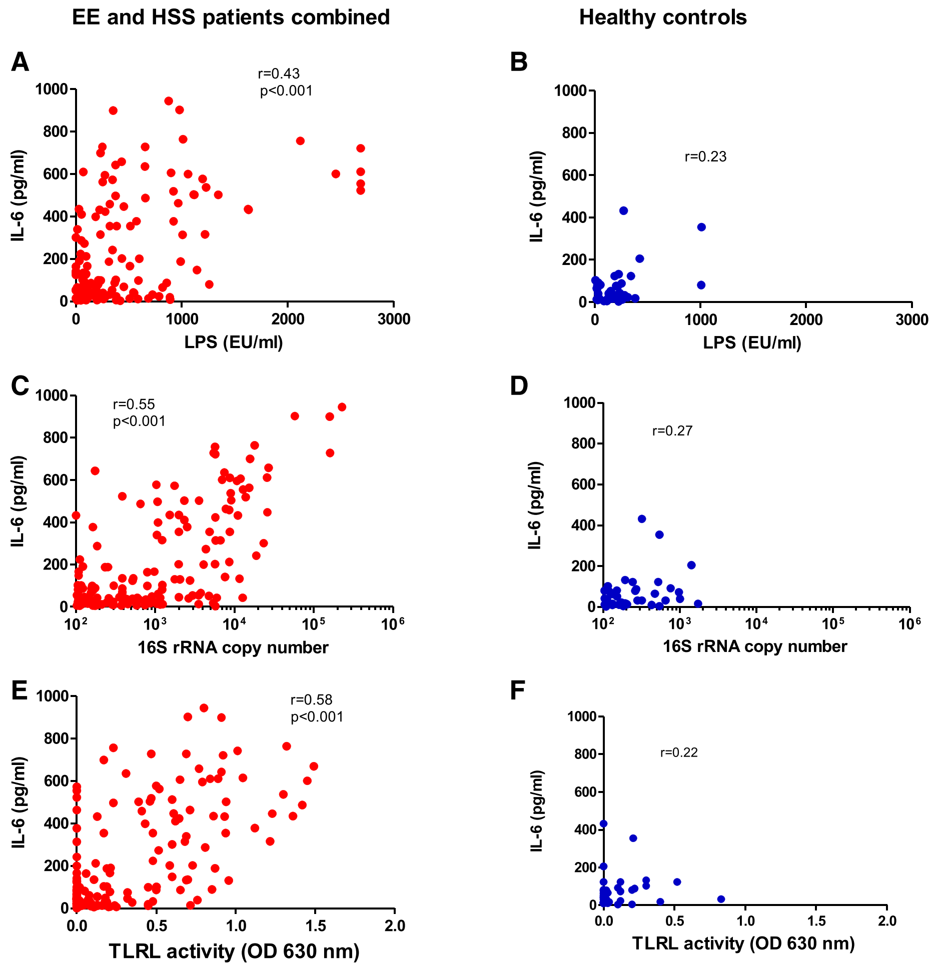

Supplemental Figure 2. Relationship between interleukin (IL)-6 and direct biomarkers of microbial translocation. Environmental enteropathy and hepatosplenic schistosomiasis patients combined correlations between IL-6 and lipopolysaccharide (LPS) (Panel **A**), IL-6 and 16S rRNA copy number (Panel **C**), and IL-6 and Toll-like receptor ligand (TLRL) activity (Panel **E**) and in healthy controls between IL-6 and LPS (Panel **B**), IL-6 and 16S rRNA copy number (Panel **D**), and IL-6 and TLRL activity (Panel **F**). Spearman correlations are shown.

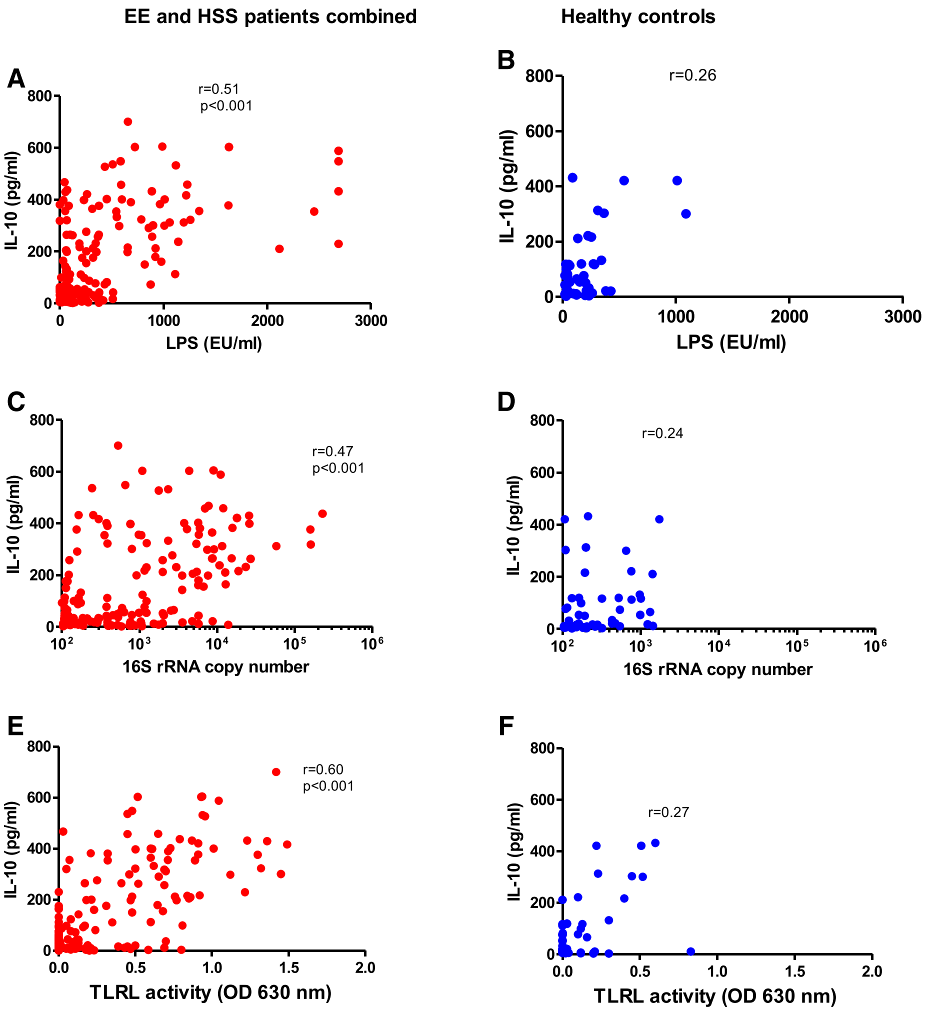

Supplemental Figure 3. Relationship between interleukin (IL)-10 and direct biomarkers of microbial translocation. Environmental enteropathy and hepatosplenic schistosomiasis patients combined correlations between IL-10 and lipopolysaccharide (LPS) (Panel **A**), IL-10 and 16S rRNA copy number (Panel **C**), and IL-10 and TLRL activity (Panel **E**) and in healthy controls between IL-10 and LPS (Panel **B**), IL-10 and 16S rRNA copy number (Panel **D**), and IL-10 and TLRL activity (Panel **F**). Spearman correlations are shown.
